# Circular RNA FAM114A2 suppresses progression of bladder cancer via regulating ∆NP63 by sponging miR-762

**DOI:** 10.1038/s41419-020-2226-5

**Published:** 2020-01-22

**Authors:** Tianyao Liu, Qun Lu, Jin Liu, Shangxun Xie, Baofu Feng, Wenjie Zhu, Minghui Liu, Yanqing Liu, Xinyan Zhou, Wu Sun, Yujing Zhang, Xi Chen, Feng Fang, Hongqian Guo, Rong Yang

**Affiliations:** 1 Department of Urology, Drum Tower Hospital, Medical School of Nanjing University, Institute of Urology, Nanjing University, Nanjing, China; 2 Jiangsu Engineering Research Center for microRNA Biology and Biotechnology, State Key Laboratory of Pharmaceutical Biotechnology, School of Life Sciences, Nanjing University, Nanjing, China; 3 The Comprehensive Cancer Centre of Drum Tower Hospital, Medical School of Nanjing University, Nanjing University, Nanjing, China; 4 Department of Pharmacology, Nanjing Medical University, Nanjing, China

**Keywords:** Prognostic markers, Bladder cancer

## Abstract

Numerous evidences have shown that circular RNAs (circRNAs) play a key role in regulating the pathogenesis of cancer. However, the mechanism of circRNAs in urothelial carcinoma of bladder (UCB) remains largely unclear. In this study, we found circFAM114A2 was significantly downregulated both in UCB tissue specimens and cell lines, and the expression level was highly correlated with pathological TNM stage and grade. Functionally, overexpression of circFAM114A2 dramatically inhibited the migration, invasion and proliferation of UCB cells in vitro, and suppressed tumor growth in vivo. Mechanistically, we confirmed miR-762 was copiously pulled down by circFAM114A2 in 5637 and T24 cells. Fluorescence in situ hybridization (FISH) further indicated the cytoplasmic interactions between circFAM114A2 and miR-762. By using luciferase reporter assay, we found that miR-762 could directly target TP63. Subsequently, we found that circFAM114A2 might increase the expression of ∆NP63 (main isoform of TP63 in UCB) by sponging miR-762. Taken together, our results demonstrated that circFAM114A2 might serve as a competing endogenous RNA (ceRNA) of miR-762 in regulating the expression of ∆NP63, thus suppressed UCB progression through circFAM114A2/miR-762/∆NP63 axis.

## Introduction

Urothelial carcinoma of bladder (UCB) is the most commonly malignant tumor of genitourinary system with high recurrence and death rates^1^. The 2015 China cancer statistics estimated that about 80,500 new cases and 32,900 deaths were caused by UCB, and the number of incidence and mortality is increasing steadily^2^. While 70–80% of UCB patients were primarily diagnosed with nonmuscle-invasive bladder cancer (NMIBC), around 50–70% will recur within 5 years, and 10–30% will develop MIBC, which showed a poor prognosis and lower 5-year survival rate^3,4^. Although surgery and chemotherapy methods are continuously being improved, the identification of novel biomarkers and effective targets for the diagnosis and therapy of UCB is still in a great desire. As the first step, much effort should be made to dissect the underlying molecular mechanisms that cause UCB. In support of this, a great number of transcripts involved in tumorigenesis and progression are proven to be dysregulated in bladder cancers, including circular RNAs (circRNAs)^5,6^.

circRNAs are a novel type of noncoding small RNAs with the character that form covalently closed loop structure in 3′ and 5′ terminus, which were first confirmed in eukaryotic cells in 1970s^7^. circRNAs originated from “exon skipping” and “direct back splicing” of pre-mRNA transcripts^8,9^, and have once considered as junk-products of splicing errors^10^. With the development of high-throughput sequencing and bioinformatical analysis, more than 30,000 circRNAs have been identified from mammalian cells^11^, which possess obviously stability, high abundance and conservation compare with liner RNAs^12,13^. These salient features reveal that circRNAs are not non-function small molecules, and may have potential roles of regulation in gene expression^14^. One of the roles has been identified to serve as competing endogenous RNAs (ceRNAs) of miRNAs^15^.

MicroRNAs (miRNAs) are a class of short noncoding RNAs with the length of approximately 23 nucleotides^16^, which regulate genes expression through directly degradation or inhibiting translation of mRNA. Increasing evidence revealed that miRNAs are dynamic, exhibiting different expression level in different diseases, and closely related to oncogenesis, progression, and metastasis in bladder cancer^17,18^. In recent years, a few studies validated that circRNAs could regulate tumorigenesis, progression, and metastasis of many malignant tumors through sponging miRNAs. For example, ciRS-7 regulated the aggression of colorectal cancer by sponging miR-7^19^. circMTO1 suppressed hepatocellular carcinoma progression through sponging oncogene miR-9 to promote the expression of p21^20^. circGFRA1 regulated GFRA1 expression via serving as sponge of miR-34a in breast cacner^21^. However, there are limited studies about the functions of circRNAs in UCB.

In the present study, we demonstrated a newly screened circRNA in UCB, circFAM114A2, which played a vital role in the progression of UCBs. By quantitative real-time polymerase chain reaction (qRT-PCR), we found that circFAM114A2 was significantly downregulated in tumor tissues from UCB patients and 3 UCB cell lines (5637, T24, and J82), and positively correlated with the tumor pathological grade. Subsequently, functional studies discovered that circFAM114A2 inhibited proliferation, migration and invasion of bladder cancer cells in vitro. Mechanistically, circFAM114A2 might serve as ceRNA of miR-762 in regulating the expression of ∆NP63 (a tumor suppressor), thus suppressed UCB progression through a circFAM114A2/miR-762/∆NP63 axis. Our study shed lights onto the design of novel therapeutic targets to treat UCB.

## Results

### CircFAM114A2 is downregulated in muscle-invasive UCB tissues and cell lines

**Fig. 1:**
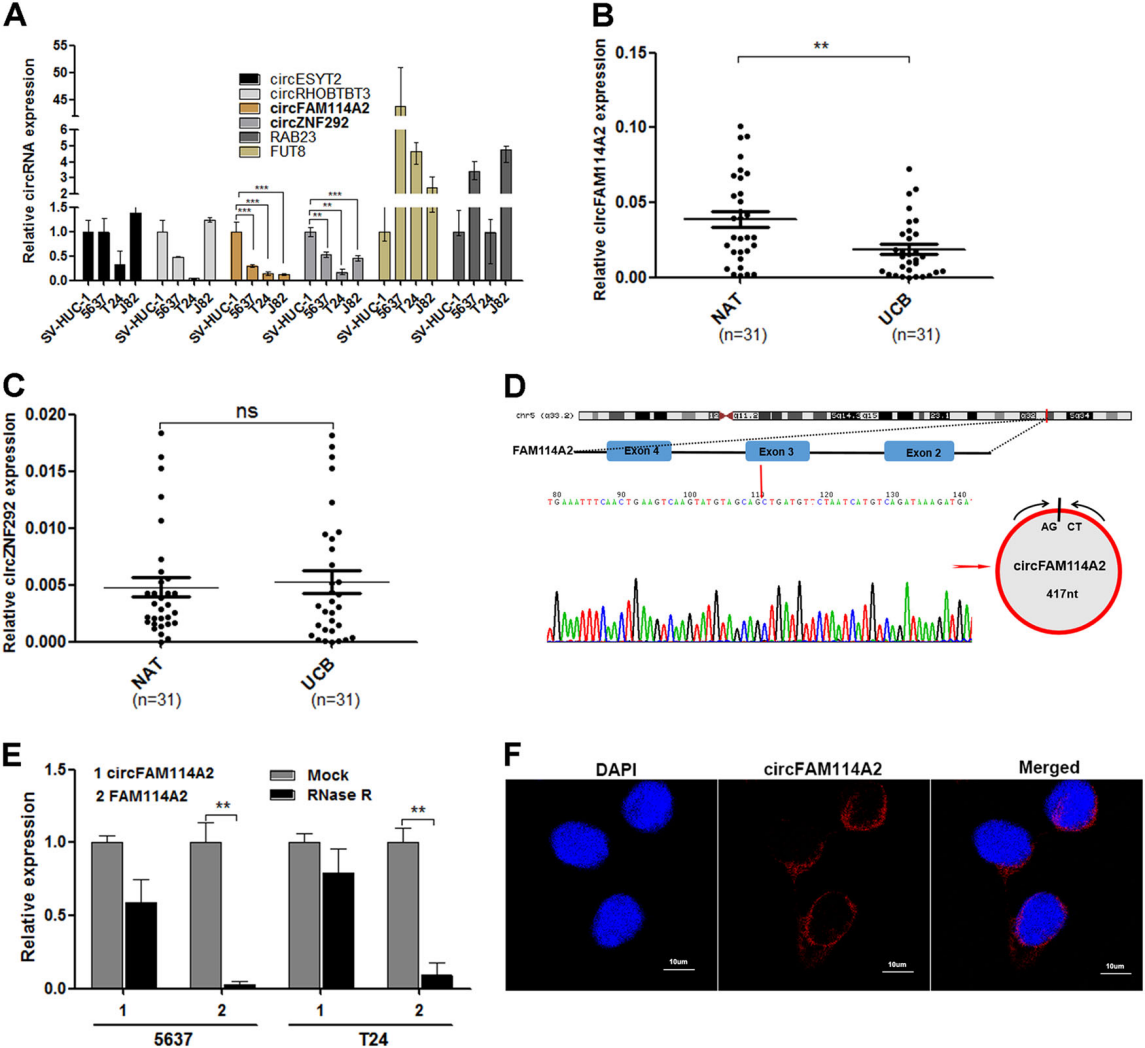
The characteristics and expression of circFAM114A2 in UCB cells. **a** The differential expression of circRNAs were measured in UCB cells by qRT-PCR. GAPDH was used as internal reference. **b**, **c** The expression level of circFAM1142 and circZNF292 were detected by qRT-PCR in 31 pairs of UCB tissues (UCB) and adjacent noncancerous tissues (NAT). GAPDH was used as internal reference. **d** Schematic illustration shows that circFAM114A2 derives from 2 to 4 exons of FAM114A2. The covalently closed structure of circFAM114A2 was validated by sanger-seq in RT-PCR products. The red arrow represents “head to tail” connection position. **e** The mRNA level of circFAM114A2 and FAM114A2 were tested by qRT-PCR in 5637 and T24 cells, which were treated with or without RNase R. GAPDH was used as internal reference. **f** RNA FISH identified that circFAM114A2 predominately localized in cytoplasm. circFAM114A2 probe was labeled with cy3, and nuclei were stained with DAPI. Scale bars, 10 µm. Date are mean ± SEM, *n* = 3. ***p < 0.001, **p < 0.01 (student’s *t* test).

To investigate the biological role of circRNAs in UCB, we selected six significantly downregulated circRNAs (fold change ≥ 3.5) from published RNA-seq data of UCB sample tissues^22^, according to the rank order of RNA-seq results. The level of these six circRNAs in UCB was confirmed by qRT-PCR in three UCB cell lines and one normal urothelial cell line. Among six circRNAs, only circFAM114A2 and circZNF292 were consistently downregulated in three cancer cell lines compared with SV-HUC-1 (Fig. 1a). Subsequently, we analyzed 31 pairs of UCB specimens and adjacent noncancerous tissues by qRT-PCR. circFAM114A2 was significantly downregulated in more than 80% bladder cancer tissues compared with adjacent normal tissues, while circZNF292 showed no meaningfully expressional difference (Fig. 1b, c). In addition, clinicopathologic features analysis revealed that low expression of circFAM114A2 was highly correlated with the pathological TNM stage and grade, but not with age, gender, tumor size, and metastasis (Table 1).

**Table 1:** The relationship between clinicalopatholgical features and circFAM114A2 expression in 31 UCB patients.

Parameters	No. (%)	CircFAM114A2 expression		
		Low (%)	High (%)	*p* Value
*Gender*				
Male	29	25	4	0.178
Female	2	1	1	
*Age*				
<70	13	11	2	0.924
≥70	18	15	3	
*Pathological stage*				
pTa–PT1	5	1	4	<0.001*
pT2–pT4	26	25	1	
*Grade*				
Low	3	1	2	0.012**
High	28	25	3	
*Tumor size*				
<3 cm	12	9	3	0.286
≥3 cm	19	17	2	
*Metastasis*				
Yes	11	9	2	0.818
No	20	17	3	
Total	31	26	5	

*Low*, lower than adjacent noncancerous tissues; *High*, higher than adjacent noncancerous tissues

*p < 0.001, chi-square test

**p < 0.05, chi-square test

Next, we obtained the detailed information of circFAM114A2 from the bioinformatics database circBase. CircFAM114A2 (hsa_circ_0001546) originated from FAM114A2 gene locates on chr5:153413351–153414527, and generated by exons 2–4 splicing with the length of 417nt. To confirm the circular characterization of circFAM114A2, divergent primer (TTGGCTGGCTCACAGTTTCC) was designed to amplify the junction area of circRNA in total RNA, which harvested from T24 and 5637 cells. Subsequently, the circFAM114A2 RT-PCR products were used to perform sanger sequencing. The sequences were head-to-tail splicing and consistent with circBank database (Fig. 1d). To test its circular characteristics, we divided the total RNA into two groups before qRT-PCR: one was pretreated with RNase R (3′–5′ exoribonuclease), and the other was used as control. It showed that linear RNA FAM114A2 was notably decreased compared with control, while circFAM114A2 was resisted to RNase R digestion (Fig. 1e). In addition, circFAM114A2 was found to be predominately localized in the cytoplasm by fluorescence in situ hybridization (FISH) (Fig. 1f). Taken together, circFAM114A2 may have potential biological roles in posttranscriptional regulation in the UCB pathophysiology.

### CircFAM114A2 inhibits the migration, invasion, and proliferation of UCB cells in vitro

**Fig. 2:**
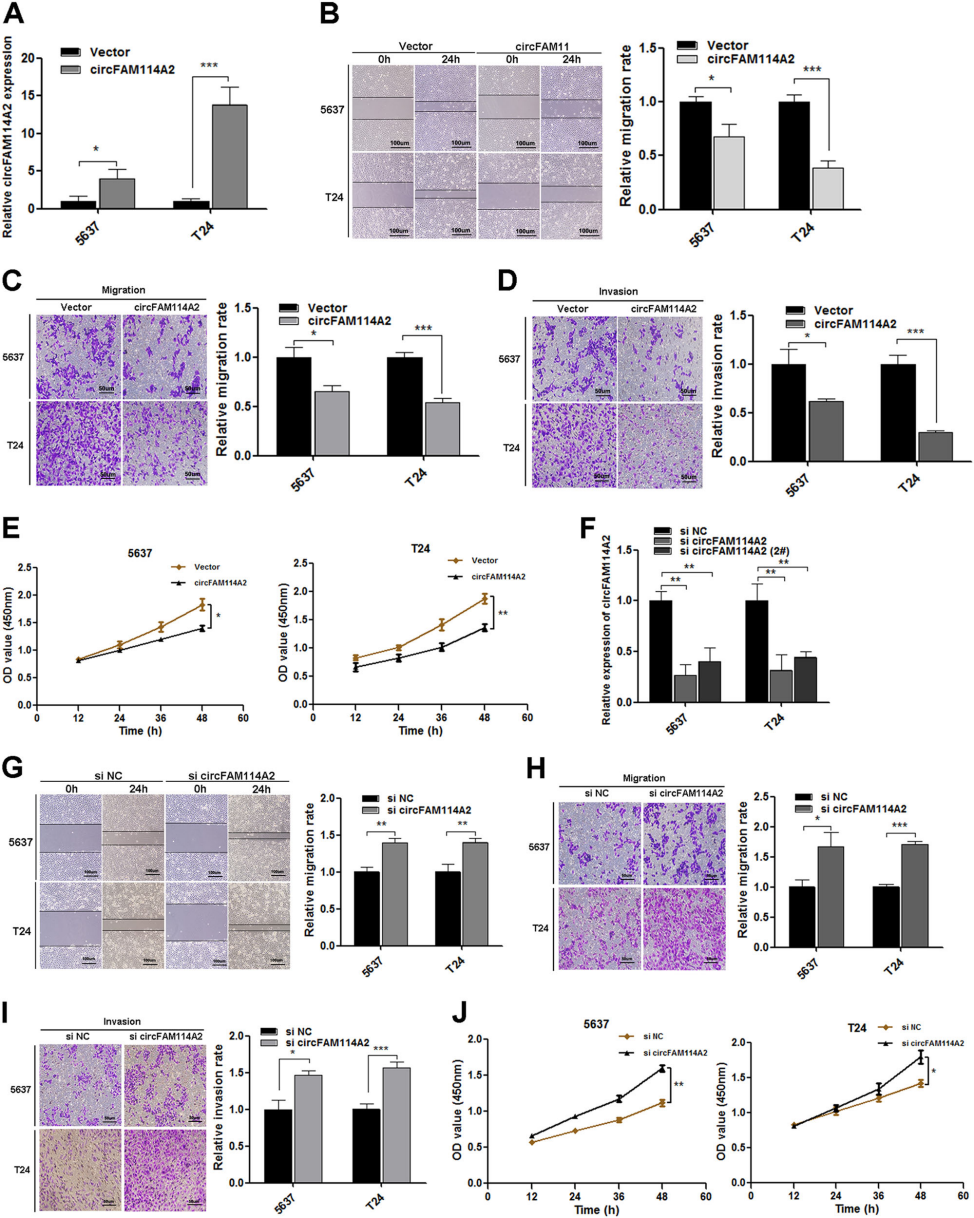
Overexpression of circFAM114A2 suppressed migration and invasion of UCB cells in vitro. **a** The expression of circFAM114A2 was measured by qRT-PCR in 5637 and T24 after transfected overexpression plasmid. **b**–**e** The biological role of circFAM114A2 on cell migration, invasion and proliferation capability were assessed by wound healing, transwell migration, and matrigel invasion assay and CCK-8 assays in 5637 and T24. **f** The expression level of circFAM114A2 in 5637 and T24 cells after knocking-down by si-circFAM114A2. **g**–**j** The effect of si-circFAM114A2 on cell migration, invasion, and proliferation ability were assessed by wound healing, transwell migration, and matrigel invasion assays and CCK-8 assay in 5637 and T24. In wound healing assay, scale bars, 100 µm. In transwell assay, scale bars, 50 µm. Date are mean ± SEM, *n* = 3. ***p < 0.001, **p < 0.01, *p < 0.05 (student’s *t* test).

After transfection of the circFAM114A2 plasmid into 5637 and T24 cells, the level of circFAM114A2 increased notably (Fig. 2a). The capabilities of migration (transwell assay), invasion (Matrigel invasion assay) and proliferation (CCK-8 assay) of 5637 and T24 cells were significantly suppressed in circFAM114A2 overexpression group (Fig. 2b-e). Next, we designed siRNAs targeting head-to-tail junction region of circFAM114A2. circFAM114A2 in 5637 and T24 cells was significantly knocked down after transfection of the siRNAs. (Fig. 2f; Fig. S1A). Functional assays confirmed that migratory, invasive and proliferative capabilities of UCB cells were negatively correlated with circFAM114A2 expression (Fig. 2g–j; Fig. S1B–D). On the basis of these results, we demonstrated that circFAM114A2 inhibit migration, invasion, and proliferation of UCB cells in vitro.

### CircFAM114A2 serves as miR-762 sponge in UCB cells

**Fig. 3:**
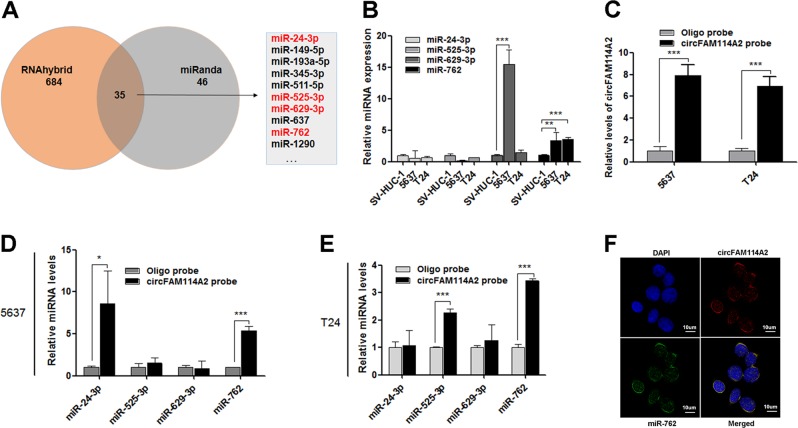
circFAM114A2 serves as sponge of miR-762 in UCB cells. **a** Schematic illustration showed the target miRNAs of circFAM114A2 predicted by RNAhybrid and miRanda. **b** The expression levels of four candidate miRNAs were detected by qRT-PCR in UCB cell lines. The level of miR-629-3P and miR-762 were increased, compared with SV-HUC-1. U6 was used as internal reference. **c** circFAM114A2 was pulled down by biotinylated probe from 5637 and T24 cells lysates, and detected by qRT-PCR. GAPDH was used as internal reference. **d**, **e** The expression levels of candidate miRNAs were assessed by qRT-PCR in circFAM114A2 pull-down products. GAPDH was used as internal reference. **f** RNA FISH showed that circFAM114A2 and miR-762 co-localized in cytoplasm. circFAM114A2 probe was labeled with cy3, miR-762 probe was labeled with FAM, and nuclei were stained with DAPI. Scale bars, 10 µm. Date are mean ± SEM, *n* = 3. ***p < 0.001, **p < 0.01, *p < 0.05 (student’s *t* test).

It has been known that circRNAs play many important roles, one of which is acting as miRNA sponge to regulate gene expression^15^. To explore miRNA sponge ability of circFAM114A2 in UCB cells, 35 miRNAs were selected from the prediction results through bioinformation analysis database (RNAhybrid and miRanda). Four miRNAs of them, as potential oncogenes, were selected as candidates (Fig. 3a). We first investigated the expression level of these miRNAs in UCB cell lines, and found that miR-629–3p and miR-762 were increased in UCB cells (Fig. 3b). Subsequently, to evaluate whether these miRNAs could be directly bound by circFAM114A2, we designed a circFAM114A2-specific probe labeled with biotin to perform pull-down assay after overexpressed circFAM114A2 in UCB cell lines. As a positive control, the level of circFAM114A2 was remarkably higher in circFAM114A2 targeted probe group than oligo probe group (Fig. 3c). Among all tested miRNAs, miR-762 was the only miRNA that was copiously pulled down in 5637 and T24 cells (Fig. 3d, e). Furthermore, circFAM114A2 and miR-762 were co-localized in cytoplasm by FISH assay (Fig. 3f). To confirm the specificity of the probes used in FISH assay, we repeated the FISH tests after transfection of si-circFAM114A2 and anti-miR-762. The results showed that the expressions of circFAM114A2 and miR-762 were significantly decreased after knockingdown (Fig. S2). These results suggested that circFAM114A2 could directly target miR-762 and function as a sponge for miR-762 in UCB cells.

### MiR-762 is overexpressed in UCB cells and tissues, and facilitates cell migration, invasion, and proliferation in vitro

**Fig. 4:**
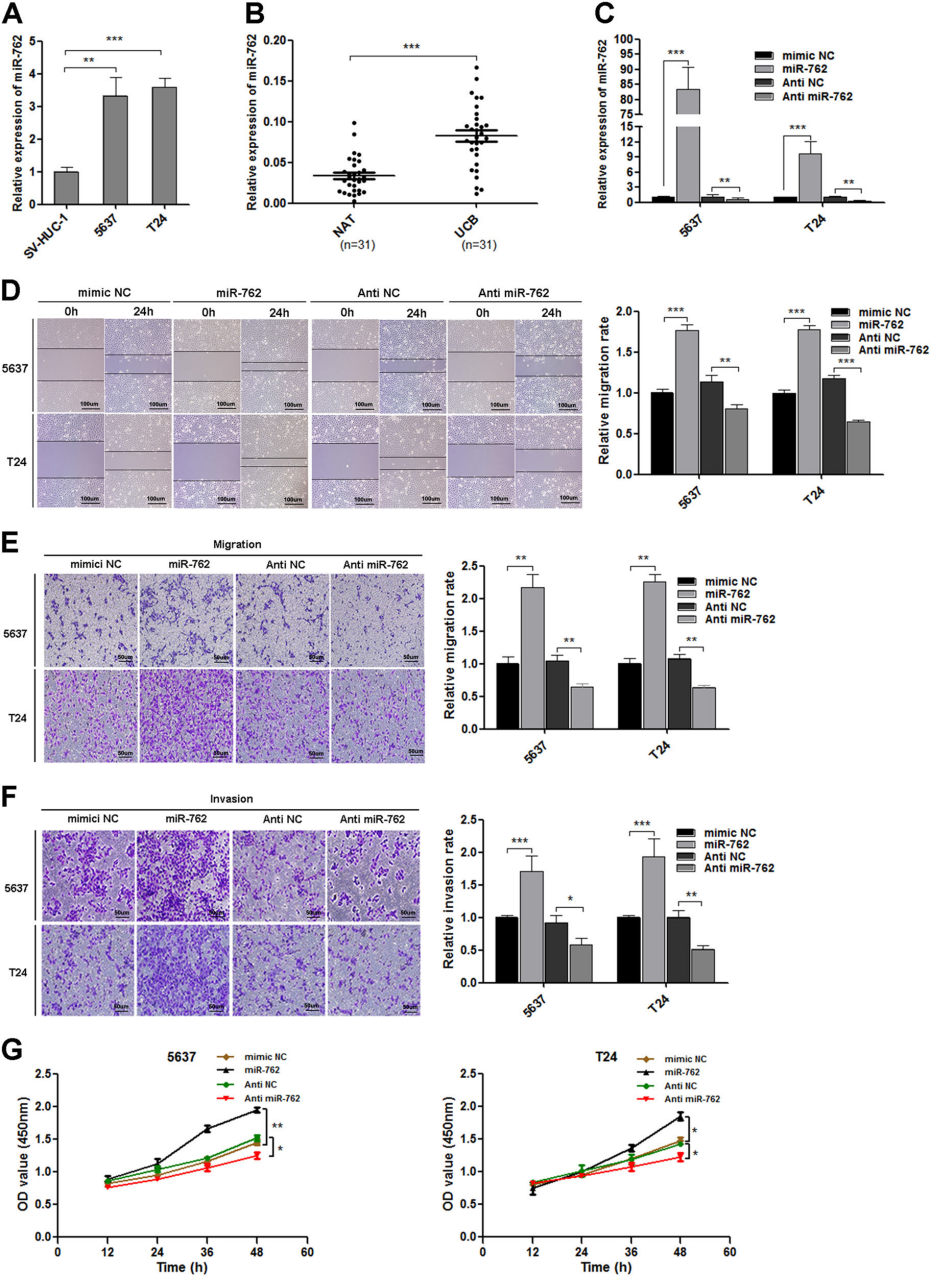
miR-762 promotes migration, invasion and proliferation of UCB cells in vitro. **a**, **b** The expression level of miR-762 was measured by qRT-PCR in UCB cells (5637, T24) and UCB tissues. miR-762 was upregulated in UCB cells and UCB tissues, compared with SV-HUC-1 cells and adjacent noncancerous tissues (NAT). U6 was used as internal reference. **c** The expression level of miR-762 was detected in 5637 and T24 cells when treated with or without miR-762 mimic or inhibitor. **d** The capability of migration was assessed by wound healing assay in 5637 and T24, which were transfected with miR-762 mimics or inhibitor. **e**–**g** The effects of miR-762 mimic and inhibitor on cell migration, invasion, and proliferation ability were assessed by transwell migration and matrigel invasion assays and CCK-8 assay in 5637 and T24. In wound healing assay, scale bars, 100 µm. In transwell assay, scale bars, 50 µm. Date are mean ± SEM, *n* = 3. ***p < 0.001, **p < 0.01, *p < 0.05 (student’s *t* test).

MiR-762 has been reported to serve as oncogene in breast cancer and ovarian cancer^23,24^. To investigate whether miR-762 plays the similar roles in UCB, we first measured the level of miR-762 in UCB cells and 31 pairs of patient tissues. miR-762 was significantly upregulated in 5637 and T24 cells compared with SV-HUC-1 (Fig. 4a). Similarly, miR-762 notably increased in majority tumor tissues compared with adjacent normal tissues (Fig. 4b). To investigate the biological function of miR-762, mimic and inhibitor of miR-762 were transfected to UCB cells. The level of miR-762 was obviously increased in mimic group, and correspondingly decreased in inhibitor group (Fig. 4c). Capabilities of migration, invasion and proliferation of UCB cells were notably enhanced after overexpression of miR-762, while it was attenuated after miR-762 knockdown (Fig. 4d–g). Taken together, our results indicated that miR-762 was an oncogene in UCB, which could promote UCB progress by facilitating cell migration, invasion, and proliferation.

### MiR-762 promotes progression of UCB via targeting △NP63

**Fig. 5:**
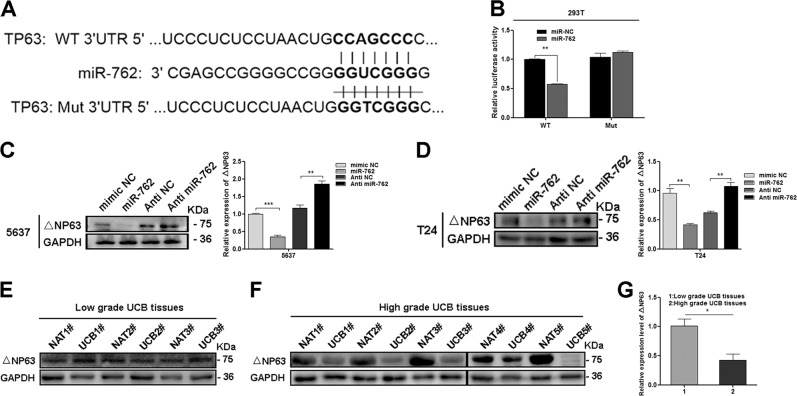
miR-762 promotes progression of UCB via targeting △NP63. **a** Targetscan prediction results shown that TP63 3′UTR contain complementary sequence of miR-762. **b** Luciferase reporter assay was performed to confirm the relationship between miR-762 and TP63/△NP63. **c**, **d** the expression level of △NP63 was detected by western blot in 5637 and T24 cells, which were transfected with miR-762 mimic and inhibitor. **e**–**g** △NP63 was at significantly lower level in high-grade UCB tissues, while not notably decreased in low grade UCB tissues, compared with noncancerous tissues. Date are mean ± SEM, *n* = 3. ***p < 0.001, **p < 0.01, *p < 0.05 (student’s *t* test).

Previous studies have revealed that circRNAs might involve in tumor progression through circRNA–miRNA–mRNA signal pathway^25^. According to the results above, we hypothesized that circFAM114A2 serves as miR-762 sponge in UCB, while the final targets still need further investigation. Using three publicly available algorithms (TargetScan, miRanda, and PicTar), TP63 was identified as a candidate mRNA that could be targeted by miR-762. TP63 is a homologous gene of the TP53 family, containing two main isoforms, TAP63 and △NP63. △Np63 is predominantly abundant compared with TAp63 in UCB^26–29^. In order to explore whether miR-762 could directly bind to the 3′UTR of TP63 mRNA, we constructed wild-type and mutant luciferase reporter plasmid of TP63 3′UTR to perform luciferase reporter assay (Fig. 5a). The luciferase activity was significantly decreased in 293T cells transfected with TP63 wild-type plasmid + miR-762 mimics, but not in the cells transfected with TP63 mutant plasmid + miR-762 mimics (Fig. 5b). Subsequently, the protein level of △NP63 (the predominant form of TP63) was decreased in 5637 and T24 cells after overexpression of miR-762. In contrast, △NP63 was significantly increased after miR-762 knockdown (Fig. 5c, d). Furthermore, the expression of △NP63 in MIBC tissues was lower than NMIBC tissues (Fig. 5e–g), consistent with previous findings that the downregulation of △NP63 closely associated with UCB progression^30,31^. All of these results indicated that TP63 is one of the downstream targeting genes of miR-762, the level of which is negatively correlated with the pathological grade of UCBs.

### CircFAM114A2 regulated the expression of △NP63 by interacting with miR-762 in vitro

**Fig. 6:**
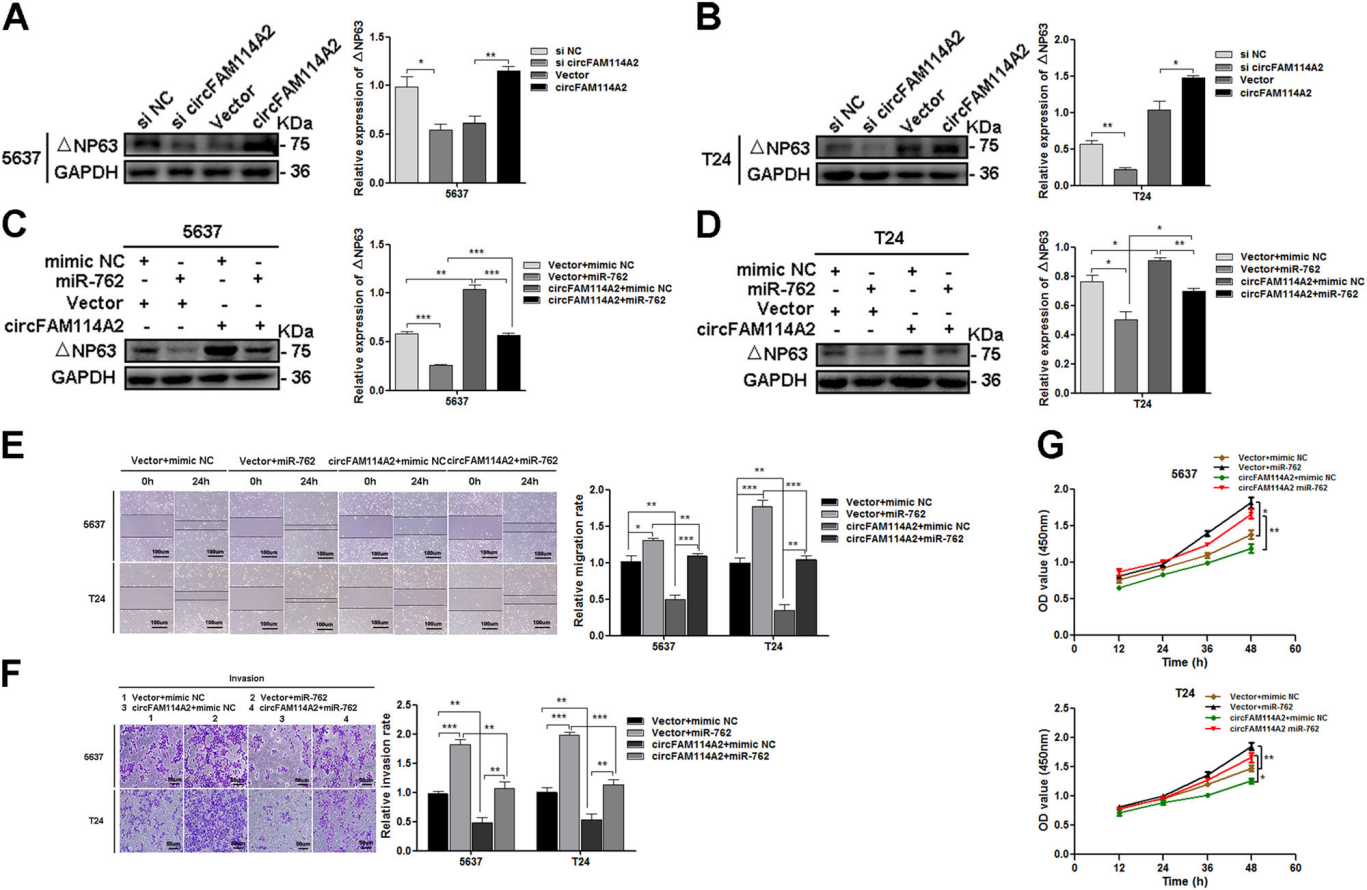
The role of circFAM114A2 targeting miR-762 in regulating the expression of △NP63 in UCB cells. **a**, **b** Overexpression of circFAM114A2 could increase the expression of △NP63, while kncokdown of circFAM114A2 displayed contrary effect. **c**, **d** miR-762 could suppress the expression level of △NP63, but the effect induced by miR-762 could be eliminated after co-transfection with circFAM114A2. **e**–**g** miR-762 could promote migration, invasion, and proliferative ability of UCB cells in vitro, but the effects were attenuated after co-transfection with circFAM114A2. In wound healing assay, scale bars, 100 µm. In transwell assay, scale bars, 50 µm. Date are mean ± SEM, *n* = 3. ***p < 0.001, **p < 0.01, *p < 0.05 (student’s *t* test).

To explore whether circFAM114A2 suppressed migration, invasion, and proliferation of UCB by regulating TP63 expression. We first examined the expression of △NP63 (the predominant isoform of TP63) in UCB cells after overexpressing or knocking down of circFAM1114A2. The level of △NP63 protein dramatically increased after circFAM114A2 was overexpressed and decreased after circFAM114A2 was knocked down in 5637 and T24 cells (Fig. 6a, b). On the other hand, when si-circFAM114A2 and △NP63 overexpression plasmids were co-transfected into UCB cells, the migratory, invasive and proliferous capabilities of UCB cells promoted by si-circFAM114A2 could be restored by overexpressing △NP63 (fig. S3). As circFAM114A2 and miR-762 have opposite effects on the level of △NP63, we speculated that circFAM114A2 regulated the expression of △NP63 by interacting with miR-762. To further confirm the interaction between circFAM114A2 and miR-762, we co-transfected circFAM114A2 plasmid and miR-762 mimic into 5637 and T24 cells. Compared to the cells transfected with Vector + miR-762 group, the level of △NP63 was significantly increased in circFAM114A2 + miR-762 group (Fig. 6c, d). Consistently, the cells transfected with both circFAM114A2 and miR-762 showed lower migratory, invasive and proliferous abilities (Fig. 6e–g). Taken together, these experiments suggested that circFAM114A2 could regulate the expression of △NP63 by sponging miR-762, and overexpressing circFAM114A2 could effectively reverse the enhancement of cell proliferation, migration, and invasion induced by miR-762.

### Overexpression of circFAM114A2 inhibited tumor growth of UCB cells in vivo

**Fig. 7:**
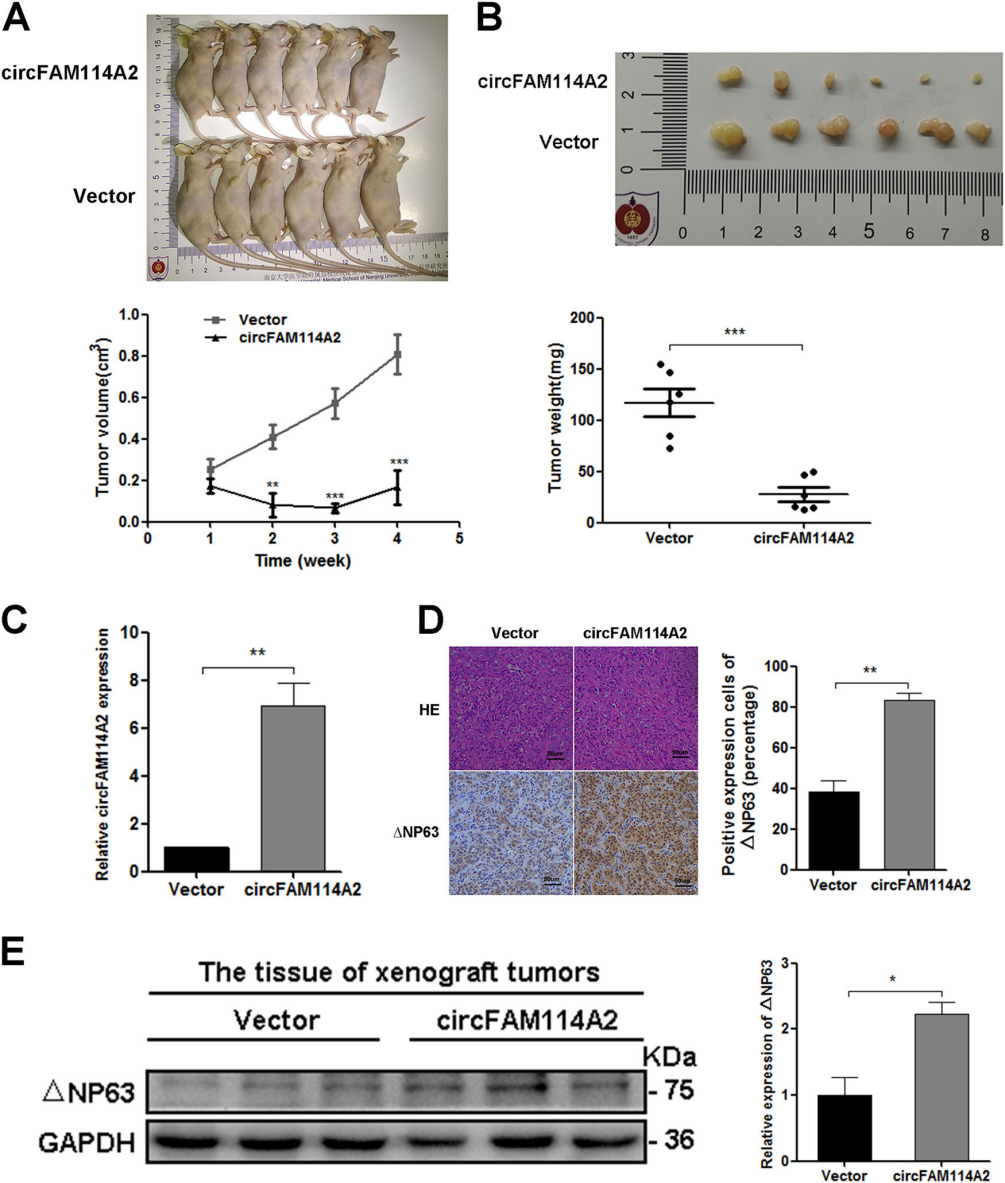
Overexpression of circFAM114A2 could inhibit the growth of UCB cells in vivo. **a**, **b** T24 cells transfected with circFAM114A2 plasmids or empty vector were subcutaneously injected into armpit of nude mice (1 × 10^7^ cells per mice, *n* = 6 each group). The growth rate and tumor weight obviously decreased in circFAM114A2 overexpression group compared with the control group. **c** The expression level of circFAM114A2 significantly increased in xenografted tumors of overexpression group, compared with the control group. **d**, **e** Immunohistochemical staining and western blot showed that overexpression of circFAM114A2 could increase the expression of △NP63. Date are mean ± SEM. Scale bars, 50 µm. ***p < 0.001, **p < 0.01, *p < 0.05 (student’s *t* test).

The expression of △NP63 in T24 cells was detected by immunofluorescence assay (IF). The results showed that △NP63 was uniformly weakly expressed in all T24 cells. After overexpressing circFAM114A2, the expression of △NP63 in T24 cells was significantly increased (Fig. S4). To further investigate the effects of circFAM114A2 in vivo, T24 cells with overexpressed circFAM114A2 or empty control vector were subcutaneously injected into armpit of BALB/c nude mice, respectively. The transfection efficiency of circFAM114A2 overexpression plasmid and the control vector could be seen in Fig. S5. The results showed that the growth rate of xenograft in circFAM114A2 overexpressed group was obviously slower than the control group (Fig. 7a). The weight of tumors was lighter in circFAM114A2 overexpressed group than control group, consistently (Fig. 7b). Subsequently, these subcutaneous tumors were collected for further study. qRT-PCR indicated that the expression of circFAM114A2 was notably higher in circFAM114A2 overexpressed group than the control group (Fig. 7c). Immunohistochemistry and western blot demonstrated that the △NP63 protein level was similarly increased in the overexpressed group (Fig. 7d, e). Taken together, these results suggested that overexpression of circFAM114A2 could suppress growth of UCB in vivo by regulating the expression of △NP63.

## Discussion

With the development of high-throughput sequencing and bioinformatics analysis, a large number of circRNAs have been discovered in various genomes and many of them were highly stable and abundantly expressed in different species. More and more evidence indicates that circRNAs can regulate gene expression and play important roles in the development and progression of multiple cancers^20,32–35^. However, the role of circRNAs in UCB is still largely unknown. In the present study, we demonstrated a new UCB related circRNA, circFAM114A2, which was found significantly downregulated in UCB tissues and cell lines. Moreover, the level of circFAM114A2 was negative correlation with the tumor grade. We further demonstrated that circFAM114A2 acts as tumor suppressor and could inhibit the proliferation and progression of UCB both in vivo and in vitro. These results revealed that circFAM114A2 may take part in the development and progression of UCB and be a potential therapeutic target.

Up to now, the studies on posttranscriptional regulation of circRNAs mainly focused on the role of miRNA sponge. CircRNAs with multiple miRNA-binding sites or miRNA response elements can serve as miRNA sponges, which had been proven to play a role in various cancers including UCB^11,15,36^. For example, circMYLK could serve as ceRNA for miR-29a and facilitate invasiveness of UCB^37^. circBCRC3 was also found to function as miR-182-5p sponge and inhibit bladder cancer proliferation^38^. However, there is still limited information about miRNA sponging function of circFAM114A2. Herein, we found that circFAM114A2 contained a binding site of miR-762 (an oncogene), and confirmed that circFAM114A2 and miR-762 co-localized in cytoplasm with RNA FISH assay. The interaction between circFAM114A2 and miR-762 in UCB was further identified by biotinylated RNA pull down. These results suggested that circFAM114A2 might exert its biological function by acting ceRNA of miR-762.

miR-762 was found to participate in the development and progression of several human cancers. For example, Li et al.^23^ found that miR-762 was upregulated both in breast cancer cell lines and tissue samples, which could effectively facilitate breast cancer cell proliferation and invasion through targeting interferon regulatory factor 7 (IRF7). However, the role of miR-762 in UCB is still unclear. In this study, we revealed that miR-762 was significantly upregulated in UCB cell lines and tissue specimens, which was negatively correlated with circFAM114A2. Functional researches indicated that miR-762 could facilitate UCB progression by promoting cell migration, invasion and proliferation of UCB. As expected, overexpressed circFAM114A2 could effectively reverse the role of migration, invasion, and proliferation contributed by miR-762.

As we all know, miRNAs regulate gene expression by sequence-selectively targeting mRNAs, leading to either translational repression or mRNA degradation. Bioinformatics prediction revealed that miR-762 could bind to 3′ -UTR of TP63 mRNA, which was a member of the p53 family of transcription factors^30^, Tp63 has two isoforms, termed TAp63 and ΔNp63. According to former studies, ΔNp63 is the major isoform present in UCB cell lines and primary tumors^31^. Although the role of ∆NP63 in bladder cancer development is still controversial, more and more studies suggested ∆NP63 was tumor suppressor in progression of UCB. Gaya et al. showed ΔNp63 expression was a favorable prognostic factor in clinically high-grade T1 bladder cancer. ΔNP63 has been found to be lost during tumor progression and proposed to be able to suppress metastasis, recently. Urist et al.^39^ also validated that ∆NP63 expression was inversely correlated with pathological grade of UCB and significantly downregulated in MIBC^26^. In present study, our data showed that ∆NP63 was notably diminished in high-grade MIBC compared with adjacent noncancer tissues and was not obviously decreased in low-grade papillary NIMBC. We also experimentally validated the inhibition of ∆NP63 translation by miR-762 through overexpressing and knocking down miR-762 in bladder cancer cells. In addition, we showed that in cultured bladder cancer cells, miR-762 inhibited ∆NP63 expression and also promoted cell proliferation and invasion. Furthermore, we found that overexpressing circFAM114A2 could restore the increased expression of ∆NP63 by miR-762 and subsequently suppress cell migration, invasion, and proliferation of UCB. Basis on these results, we demonstrated that circFAM114A2 serves as a miRNA sponge to inhibit the progression of UCB through the circFAM114A2/miR-762/∆NP63 axis (Fig. S6).

Besides acting as a miRNA sponge to repress miRNA function, recent studies have shown that circRNAs can also participate in splicing target genes, translate genes into protein and interact with RNA-binding proteins. Other roles of circFAM114A2 in bladder cancer are still need to be investigated in our future studies.

## Conclusion

In summary, circFAM114A2 could serve as a ceRNA of miR-762 in regulating the expression of ∆NP63, thus suppressed UCB progression through the circFAM114A2/miR-762/∆NP63 axis. Thus, our results suggest that circFAM114A2 might serve as a promising potential biomarker and a novel therapeutic target for UCB.

## Materials and methods

### Clinic patients’ tissue specimens

The bladder cancer tissues and paired adjacent noncancer tissues were obtained from the patients who underwent radical cystectomy or transurethral resection of bladder tumor at the Affiliated Drum Tower Hospital of Nanjing University, School of Medicine (Nanjing, China). All tissue specimens were immediately frozen in liquid nitrogen and then stored at −80 °C refrigerator after surgical resection. All the patients provided paper informed consent, and this study was approved by the Ethics Committee of Nanjing University. Clinical features of the patients are presented in Table 1.

### Cell culture and treatment

All cell lines (T24, J82, 5637, SV-HUC-1, and 293T) were purchased from the Shanghai Institute of Cell Biology at the Chinese Academy of Sciences (Shanghai, China) with authenticated using short tandem repeat profiling, tested for mycoplasma contamination. T24, J82, 5637, and SV-HUC-1 were cultured in RPMI 1640 medium supplemented with 10% fetal bovine serum (FBS, Genial, South America Origin). 293T cells were cultured in Dulbecco’s modified Eagle’s medium supplemented with 10% FBS. All cells were incubated in a humidified atmosphere at 37 °C with 5% CO_2_.

### RNA extraction and qRT-PCR

Total RNA was extracted from the cultured cells and tissues using Trizol Reagent (Invitrogen, CA, USA) according to the manufacturer’s instructions. For RT-PCR, complementary DNA was synthesized with 1 μg total RNA using Prime Script RT Master Mix (Takara, Japan). To quantify the amount of circRNA, miRNA and mRNA, the real-time PCR analyses were performed using SYBR Green Premix Ex Taq^TM^ kit (Takara) in Biosystems 7500 Sequence Detection System (Applied Biosystems). All of the reactions were performed in triplicate. After the reactions were completed, the cycle threshold (CT) data were determined using fixed threshold settings, and the mean CT was determined from triplicate PCRs. A comparative CT method was used to compare each condition to the control reactions. In miRNA RT-PCR reaction, U6 was used as an internal control, and the relative level of miRNA normalized with U6 was calculated with the equation 2^−ΔΔCT^ in which ΔΔCT = (CT_miR-762_ − CT_U6_)_tumor_ − (CT_miR-762_ − CT_U6_)_control_. For circRNA and mRNA, GAPDH was used as an internal control. The calculation method is similar to that described above. The primer sequence was listed in Table S1.

### RNase R treatment

Total RNA was extracted from 5637 and T24 cells, and divided into two groups. One group was pretreated with RNase R (Geneseed, Guangzhou, China), 3 U/µg RNA, 37 °C, for 30 min according to the manufacturer’s instructions. The rest was used as control. Then qRT-PCR was performed to detect the expression of circFAM114A2 and FAM114A2 with/without RNase R treatment.

### Overexpression plasmids construction and siRNA interference assay

Human circFAM114A2 and △NP63 overexpression plasmids were purchased from Genomeditech (Shanghai, China) and Obio Technology Corp. Ltd. (Shanghai, China), respectively. Empty vector plasmid was used as negative control. The siRNAs sequence with specifically interfering effect (si-circFAM114A2) was purchased from GenePharma (Shanghai, China),and a scrambled siRNA as negative control. The circFAM114A2 overexpression plasmid and siRNAs were transfected into T24 cells and 5637 cells using Lipofectamine 2000 (Invitrogen), according to the manufacturer’s instructions. Total RNA and protein were extracted 48 h after transfection and assessed by qRT-PCR and western blot, respectively. The siRNA sequence was listed in Table S2.

### Oligonucleotide synthesized and transfection

miRNA mimics and inhibitors were designed and synthesized by Ribobio (Guangzhou, China), and transfection into T24 and 5637 cells with Lipofectamine 2000 (Invitrogen) according to the manufacturer’s instructions. Transfection efficiency was assessed by qRT-PCR. The sequences of miRNA mimics and inhibitors were listed in Table S3.

### Wound healing assay

T24 and 5637 cells were seeded in 6-well plates with 5 × 10^5^ cells per well. Overexpression plasmids or oligonucleotides were transfected when cell confluency reached 80%. Twenty-four hour after transfection, a wound was made by the fine end of 200 μl pipette tip. Photographs were taken at the appropriate time to estimate the area occupied by migratory cells.

### Transwell migration and invasion assays

For transwell assay, a transwell chamber (Costar, New York, NY, USA) with or without precoated matrigel (BD Science, USA) was used to test the invasion and migration capacities of bladder cancer cells (5637 and T24) in vitro. Cells suspended in 200 μl serum-free medium were inoculated in the transwell upper chambers (5 × 10^4^ cells/well for migration, 1 × 10^5^ cells/well for invasion), and 500 μl medium supplemented with 10% FBS was added to the lower chambers. After incubation (24 h for migration and 48 h for invasion) in a humidified atmosphere at 37 °C with 5% CO_2_, cells in the upper chamber were wiped off with a cotton swab, and cells in the lower membrane surface were fixed with 4% paraformaldehyde and stained with 0.5% crystal violet. The number of migrated and invaded cells were counted in three randomly selected fields.

### Cell counting kit-8 assay

The proliferation capability of UCB cells (5637 and T24) were assessed by cell counting kit-8 assay (CCK-8) according to the manufacturer’s instructions. The transfected cells were seeded into 96-well plates (5 × 10^3^ cells/well). At 12, 24, 36, and 48 h after transfection, 10 μl of CCK-8 reagent was added to the test well and incubated for 2 h. The absorbance was measured at a wavelength of 450 nm.

### Fluorescence in situ hybridization

When cell confluency reached 70–80%, the bladder cancer cells were fixed with paraformaldehyde, and then prehybridized and hybridized in hybridization buffer. Cy3-labeled circFAM114A2 targeted probes (5′-ACTTCAGTTCATACATCGTCGACTACAAGATTAGTACAGTC-3′) and fam-labeled miR-762 targeted probes (5′-GGGGCTGGGGCCGGGGCCGAGC-3′) were designed and synthesized by GenePharma (Shanghai, China). The signals of the probe were detected by Fluorescent In Situ Hybridization Kit (GenePharma, China) according to the manufacturer’s instruction. Nuclei were stained with DAPI. The images were captured on TCS SP5II confocal microscope (Leica Microsystems, Mannheim, Germany).

### MiRNA targets prediction of circFAM114A2

We predicted the specifically binding sites of circFAM114A2 interacting with miRNA and miRNA targeting downstream gene by using bioinformatic datebase RNAhybrid (https://bibiserv.cebitec.uni-bielefeld.de/rnahybrid/), miRanda (http://www.microrna.org/microrna/getMirnaForm.do), and TargetScan (http://www.targetscan.org).

### Pull-down assay

For pull-down assay, the biotinylated circFAM114A2 probe and oligo probe (used as negative control) were designed and synthesized by Tsingke (Tsingke, Wuhan, China). Approximately, 1 × 10^7^ cells were harvested and lysed after transfection with overexpressed plasmid. The biotinylated circFAM114A2 probe was incubated with streptavidin magnetic beads (Life Technologies, USA) at room temperature (RT) for 2 h to generate probe-coated beads. Then cell lysates were incubated with probe-coated beads at 4 °C overnight. The beads were subsequently washed. The RNA was extracted with Trizol (Invitrogen) and assessed by qRT-PCR. The sequence of circFAM114A2 probe was: 5′-TAGAACATCAGCTGCTACATACTTG-3′. That of oligo probe was 5′-GTGTAACACGTCTATACGCCCA-3′.

### Luciferase reporter assay

A fragment of TP63 3′-UTR containing miR-762 binding site was inserted into a luciferase reporter plasmid (Realgene, Nanjing, China), and used to test the direct binding of miR-762 to the target gene TP63. The insertion was confirmed to be correct by sequencing. To test the binding specificity, the sequences that interacted with the miR-762 seed sequence were mutated (from CCAGCCC to GGTCGGGG), and the mutant TP63 3′-UTR was inserted into an equivalent luciferase reporter. 293T cells were cultured in 24-well plates, and then co-transfected with luciferase reporter plasmid, miR-762 mimic or mimic-NC and β-galactosidase (β-gal) expression plasmid (Ambion). The β-gal plasmid worked as control. The luciferase activity were measured with luciferase assay kit (Promega, Madison, WI, USA) 24 h after transfection according to the manufacturer’s instructions.

### Protein preparation and Western blot

The tissues and cells lysates were prepared with radioimmunoprecipitation assay buffer (Beyotime, Shanghai, China) supplemented with phenylmethylsulfonyl fluoride (Beyotime, Shanghai, China). The supernatant of lysates was collected after centrifugation, and the protein concentration was determined with the Pierce BCA protein assay kit (Thermo Scientific, Rockford, IL, USA) according to the manufacturer’s instructions. The Proteins were separated by 10% sodium dodecyl sulfate polyacrylamide gel electrophoresis (Bio-Rad). The △NP63 protein level was analyzed by western blot with antibody for △NP63 (poly6190, BioLegend, CA, USA). The protein levels were normalized by probing the same blots with a GAPDH antibody (FL-335, sc-25778, Santa Cruz Biotechnology, CA, USA).

### Animal experiment

All animal experiments were approved by the Institutional Review Board of Nanjing University (Nanjing, China). Four-week-old female BALB/c nude mice were purchased from the Model Animal Research Center of Nanjing University (Nanjing, China), and randomly divided into two groups (*n* = 6, respectively, no blinding was done). T24 cells transfected with circFAM114A2 plasmid or control vector were subcutaneously injected into armpit of nude mice (1 × 10^7^ cells/mice), respectively. The volume of the tumors was measured every week after implantation. The tumor volume was calculated by the following formula: tumor volume [mm^3^] = (length [mm]) × (width [mm])^2^ × 0.52. The mice were sacrificed after 28 days. The tumors were excised, and the tumor weight was measured. Portions of the tumor samples were used for protein and total RNA extraction, and the remainder was fixed in 4% paraformaldehyde for 24 h and then processed for hematoxylin and eosin (H&E) staining as well as immunohistochemical staining for △NP63.

### HE and immunohistochemistry

The tissue paraffin sections were divided into two groups: one for HE stain and the other for IHC assay. For IHC assay, paraffin sections were washed with xylene and alcohol to dewax, and subsequently repaired with citric acid antigen repair buffer (PH 6.0) for antigen repair, according to manufacturer's instruction. The sections were then incubated in 3% hydrogen peroxide solution at RT for 25 min to block endogenous peroxidase, and washed in phosphate buffer solution (PBS). Next, the sections were blocked by 5% bovine serum albumin (BSA) for 30 min, and then incubated in primary antibody of rabbit anti-△NP63 (1: 500, Servicebio, Wuhan, China) in wet box overnight at 4 °C. Then rinsed the sections in PBS (3 × 5 min, RT). After that, the sections were incubated in horseradish peroxidase secondary antibody (1:200, Servicebio, Wuhan, China) for 50 min at RT, and then washed with PBS (3 × 5 min, RT). After that, the diaminobenzidine chromogenic solution (Servicebio, Wuhan, China) was added to the sections, and used microscope to control the coloration time (the positive color was brownish yellow). Then rinsed the sections with tap water to stop the coloration. Next, nuclei were stained with hematoxylin and the sections were washed with tap water. Finally, the sections were dehydrated and sealed, and the image were captured by Olympus FSX100 microscope (Olympus, Japan).

### Immunofluorescence

For IF assay, T24 cells were cultured in 24-well plate. After treatment, the cells were fixed with 4% paraformaldehyde for 15 min at RT, and then washed in PBS for 3 × 3 min. Next, the cells were permeabilized and blocked using 5% BSA (Sigma) and 0.5% Triton X-100 in PBS (PBST) for 1 h at RT, and then incubated with rabbit anti-△NP63 (E6Q3O, CST, USA) in 5% BSA overnight at 4 °C. After removing primary antibody, the cells were washed in PBST for 3 × 3 min, and then incubated in secondary fluorescent antibody (488 nm, Thermo Fisher Scientific, MA, USA) in 5% BSA in a light-proof environment for 1 h at RT. Next, the cells were stained with DAPI (Beyotime, Shanghai, China) in a light-proof environment for 10 min at RT. Finally, the cells were washed with PBS (3 × 5 min, RT) and visualized using a confocal microscope (Leica Microsystems, Mannheim, Germany).

### Statistical analysis

Sample size was chosen based on the need for statistical power. All results were representative of at least three independent experiments. The variance between the groups that are being statistically compared is similar. qRT-PCR and the luciferase reporter were performed in triplicate, and each individual experiment was repeated three times. In our study, unless otherwise noted, all data were shown as mean ± SEM, and experimental data were evaluated by *t* test (unpaired, two-tailed). The chi-square test was used for analysis the correlation between clinicopathological features of patients with UCB and circFAM114A2 expression profiles. These statistical analyses were performed using Graphpad Prism statistical software (Version 5; La Jolla, CA, USA) and SPSS 25.0 statistical software package. *p < 0.05 was considered statistically difference.

## Supplementary information

Supplementary information

Primer sequences

siRNA sequences

The sequences of Oligonucleotide

The effect of si-circFAM114A2 (2#) in UCB cells.

RNA FISH showed that circFAM114A2 and miR-762 localized in cytoplasm, and the Fluorescence intensity of circFAM114A2 and miR-762 were notably decreased after knockdown.

Overexpression of ∆NP63 could restore the effect of circFAM114A2 silencing.

The expression level of ∆NP63 in T24 cells. scale bars, 100um.

The transfection efficiency of circFAM114A2 and Vector in T24 cells. scale bars, 50um.

The schematic diagram illustrates that circFAM114A2 serves as a ceRNA for miR-762, and inhibits the progression of UCB through the circFAM114A2/miR-762/∆ NP63 axis.

The uncropped versions of western blots.

The raw images of IHC of ∆ NP63 of each animal.
