# Supplementary material for: Circular RNA FAM114A2 suppresses progression of bladder cancer via regulating ∆NP63 by sponging miR-762
Source: Cell Death Dis. 2020 Jan 22;11(1):47. doi: 10.1038/s41419-020-2226-5 (PMC6976626; doi:10.1038/s41419-020-2226-5)
Supplement: Supplementary file 1 — Supplementary information [file 41419_2020_2226_MOESM1_ESM.docx]

**Supplementary files:**

**Supplementary file 1:**

**Table S1, S2 and S3** The sequences of primers and oligonucleotides used in this study.

**Supplementary file 2:**

**Figure S1.** The effect of si circFAM114A2 (2#) in UCB cells. **A** The expression level of circFAM114A2 in 5637 and T24 cells after knocking-down by si-circFAM114A2 (2#). **B-D** The effect of si-circFAM114A2 on cell migration, invasion and proliferation ability were assessed by wound healing, transwell matrigel invasion assays and CCK-8 assay in 5637 and T24. In Wound healing assay, scale bars, 100um. In transwell assay, scale bars, 50um. Date are mean ± SEM, n=3. *** p<0.001, ** p<0.01 (student’s *t*-test).

**Figure S2.** RNA FISH showed that circFAM114A2 and miR-762 localized in cytoplasm, and the Fluorescence intensity of circFAM114A2 and miR-762 were notably decreased after knock down. circFAM114A2 probe was labeled with cy3, miR-762 probe was labeled with FAM, and nuclei were stained with DAPI. Scale bars, 10um.

**Figure S3.** Over-expression of ∆NP63 could restore the effect of circFAM114A2 silencing. **A-B** the expression level of ∆NP63 could be suppressed by si-circFAM114A2, but the effect could be eliminated after co-transfection with ∆NP63. **C-E** si-circFAM114A2 could promote migration, invasion and proliferative ability of UCB cells in vitro, but the effects could be significantly attenuated after co-transfection with ∆NP63. In Wound healing assay, scale bars, 100um. In transwell assay, scale bars, 50um. Date are mean ± SEM, n=3. *** p<0.001, ** p<0.01, * p<0.05 (student’s *t*-test).

**Figure S4.** The expression level of ∆NP63 in T24 cells. scale bars, 100um.

**Figure S5.** The transfection efficiency of circFAM114A2 and Vector in T24 cells. scale bars, 50um.

**Figure S6.** The schematic diagram illustrates that circFAM114A2 serves as a ceRNA for miR-762, and inhibits the progression of UCB through the circFAM114A2/miR-762/∆ NP63 axis.

**Figure S7.** The uncropped versions of western blots.

**Figure S8**. The raw images of IHC of ∆ NP63 of each animal. The positive color was brownish yellow. Scale bars, 50um.
