# Supplementary material for: Circular RNA FAM114A2 suppresses progression of bladder cancer via regulating ∆NP63 by sponging miR-762
Source: Cell Death Dis. 2020 Jan 22;11(1):47. doi: 10.1038/s41419-020-2226-5 (PMC6976626; doi:10.1038/s41419-020-2226-5)
Supplement: Supplementary file 2 — Primer sequences [file 41419_2020_2226_MOESM2_ESM.docx]

**Table S1 Primer sequences.**

| Genes Name | Primer sequence (5’-3’) |
| --- | --- |
| circFAM114A2 | F:TGGGGCAAGTCCATACTCTC  R:TTGGCTGGCTCACAGTTTCC |
| circESYT2 | F:TGCCAAATGCGTCAAACCTC  R:AGGGGTCTGACTTTCCCTTG |
| circZNR292 | F:GGGTGTGGAAAAACCCGGTA  R:GCGTTCCAGAACCAAGGCTA |
| circRAB23 | F:CTGAGGCACTGGCAAAAAGG  R:TTGGAGCTGAAATGGTTTCTGT |
| circFUT8 | F:TTCTTTTTGCCTGGGGGACC  R:TCCTGGTGATATGTGTAGGAAGC |
| circRHOBTB3 | F:AGGCAACCCACCATTACGAG  R:TGTGACGCTTCAGCCTTTAAGA |
| miR-24-3P | F:ACACTCCAGCTGGGTGGCTCAGTTCAGCAG  R:CTCAACTGGTGTCGTGGAGTCGGCAATTCAGTTGAGCTGTTCCT |
| miR-525-3P | F:ACACTCCAGCTGGGGAAGGCGCTTCCCTTT  R:CTCAACTGGTGTCGTGGAGTCGGCAATTCAGTTGAGCGCTCTAA |
| miR-629-3P | F:ACACTCCAGCTGGGGTTCTCCCAACGTAAG  R:CTCAACTGGTGTCGTGGAGTCGGCAATTCAGTTGAGGCTGGGCT |
| miR-762 | F:ACACTCCAGCTGGGGGGGCTGGGGCCGGGG  R:CTCAACTGGTGTCGTGGAGTCGGCAATTCAGTTGAGGCTCGGCC |
| FAM114A2 | F:CTCGGAAAAGACCAGAGACCA  R:GAGGAGAGTATGGACTTGCCC |
| unified reverse primer | TGGTGTCGTGGAGTCG |
| U6 | F:CTCGCTTCGGCAGCACA  R:AACGCTTCACGAATTTGCGT |
| GAPDH | F:CAATGACCCCTTCATTGACC  R:TTGATTTTGGAGGGATCTCG |
