# Supplementary material for: Circular RNA FAM114A2 suppresses progression of bladder cancer via regulating ∆NP63 by sponging miR-762
Source: Cell Death Dis. 2020 Jan 22;11(1):47. doi: 10.1038/s41419-020-2226-5 (PMC6976626; doi:10.1038/s41419-020-2226-5)
Supplement: Supplementary file 3 — siRNA sequences [file 41419_2020_2226_MOESM3_ESM.docx]

**Table S2 siRNA sequences.**

| Gene name | Sequences |
| --- | --- |
| si-circFAM114A2 | Sense: UAUGUAGCAGCUGAUGUUCTT  Anti-sense: GAACAUCAGCUGCUACAUATT |
| si-circFAM114A2 (2#) | Sense: GUAUGUAGCAGCUGAUGUUTT  Anti-sense: CAUACAUCGUCGACUACAATT |
| si NC | Sense: UUCUCCGAACGUGUCACGUTT  Anti-sense: ACGUGACACGUUCGGAGAATT |
