# Supplementary material for: Circular RNA FAM114A2 suppresses progression of bladder cancer via regulating ∆NP63 by sponging miR-762
Source: Cell Death Dis. 2020 Jan 22;11(1):47. doi: 10.1038/s41419-020-2226-5 (PMC6976626; doi:10.1038/s41419-020-2226-5)
Supplement: Supplementary file 4 — The sequences of Oligonucleotide [file 41419_2020_2226_MOESM4_ESM.docx]

**Table S3 The sequences of Oligonucleotide.**

| Genes name | Sequences |
| --- | --- |
| mimic negative control | Sense: UUCUCCGAACGUGUCACGU  Anti-sense: AAGAGGCUUGCACAGUGCA |
| miR-762 mimic | Sense: GGGGCUGGGGCCGGGGCCGAGC  Anti-sense: CCCCGACCCCGGCCCCGCUCG |
| miR-762 inhibitor | GCUCGGCCCCGGCCCCAGCCCC |
| miR-762inhibitor negative control | ACGUGACACGUUCGGAGAA |
