# Supplementary figures and images for: Circular RNA FAM114A2 suppresses progression of bladder cancer via regulating ∆NP63 by sponging miR-762
Source: Cell Death Dis. 2020 Jan 22;11(1):47. doi: 10.1038/s41419-020-2226-5 (PMC6976626; doi:10.1038/s41419-020-2226-5)

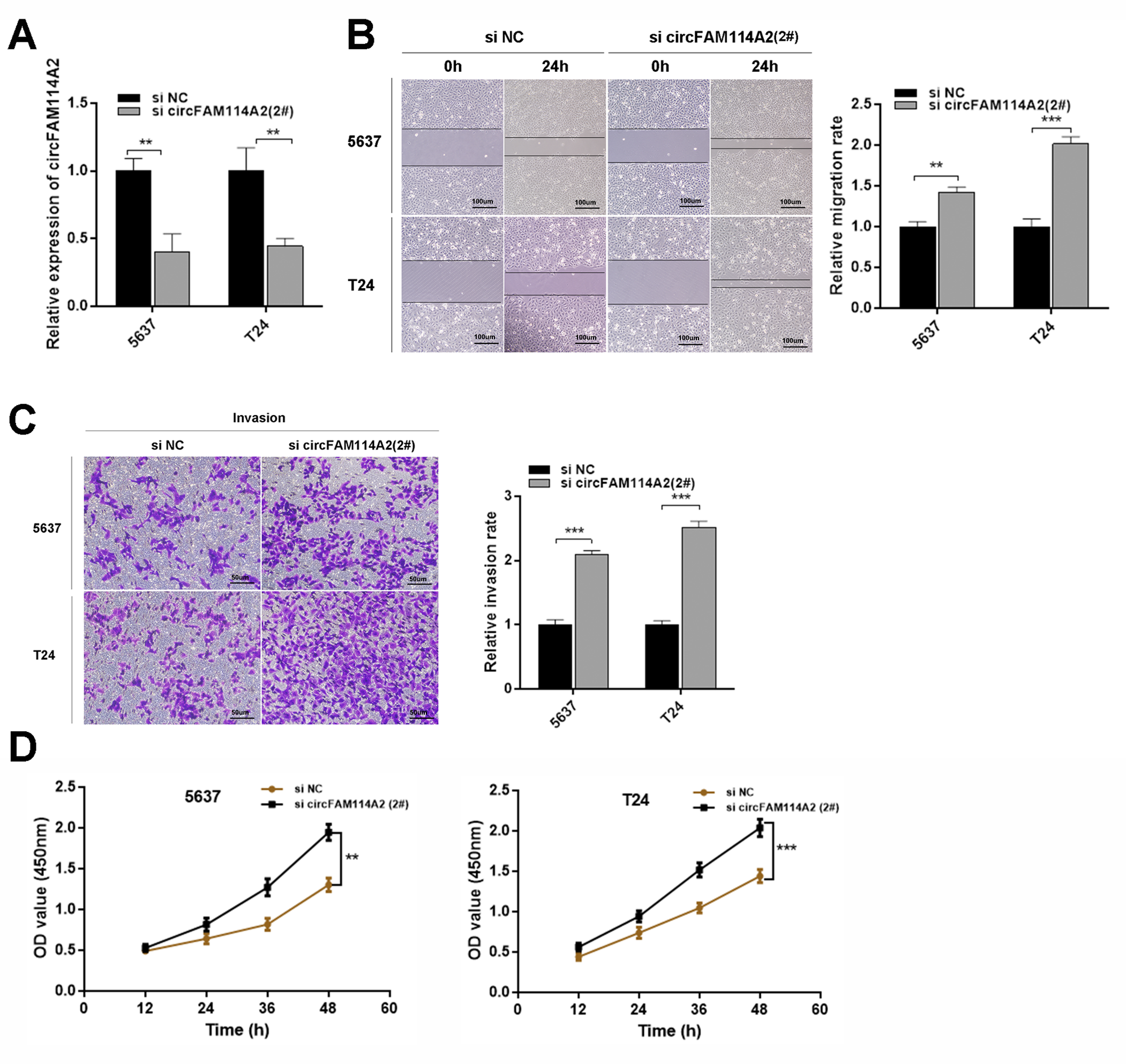

Supplement: Supplementary file 5 — The effect of si-circFAM114A2 (2#) in UCB cells. [file 41419_2020_2226_MOESM5_ESM.tif]

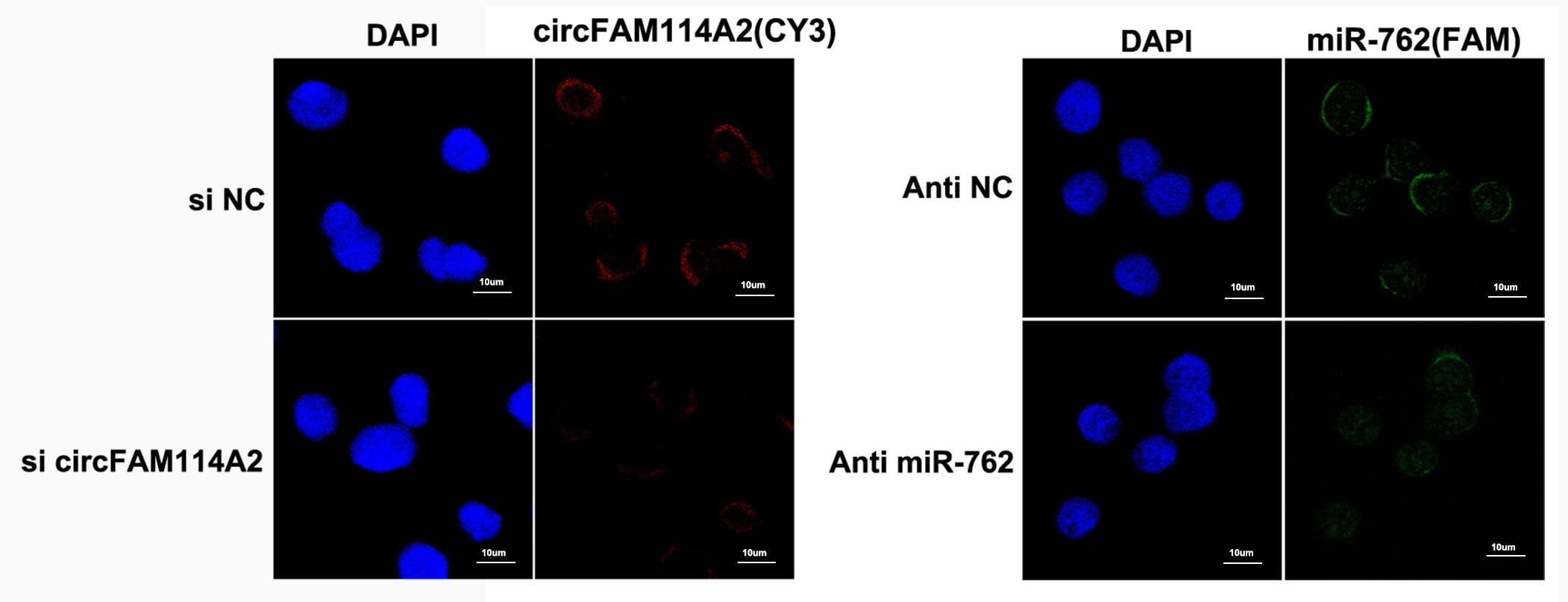

Supplement: Supplementary file 6 — RNA FISH showed that circFAM114A2 and miR-762 localized in cytoplasm, and the Fluorescence intensity of circFAM114A2 and miR-762 were notably decreased after knockdown. [file 41419_2020_2226_MOESM6_ESM.tif]

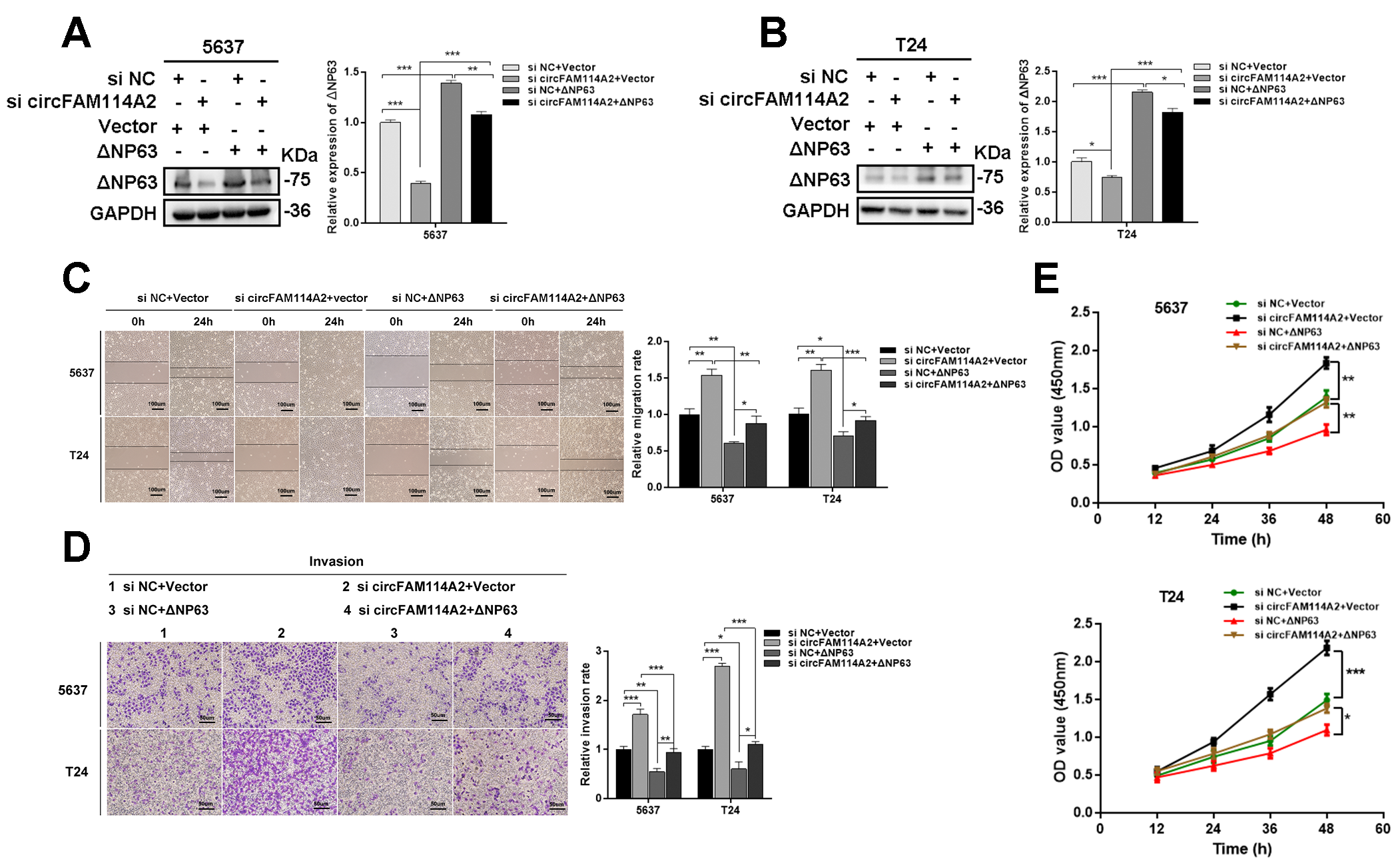

Supplement: Supplementary file 7 — Overexpression of ∆NP63 could restore the effect of circFAM114A2 silencing. [file 41419_2020_2226_MOESM7_ESM.tif]

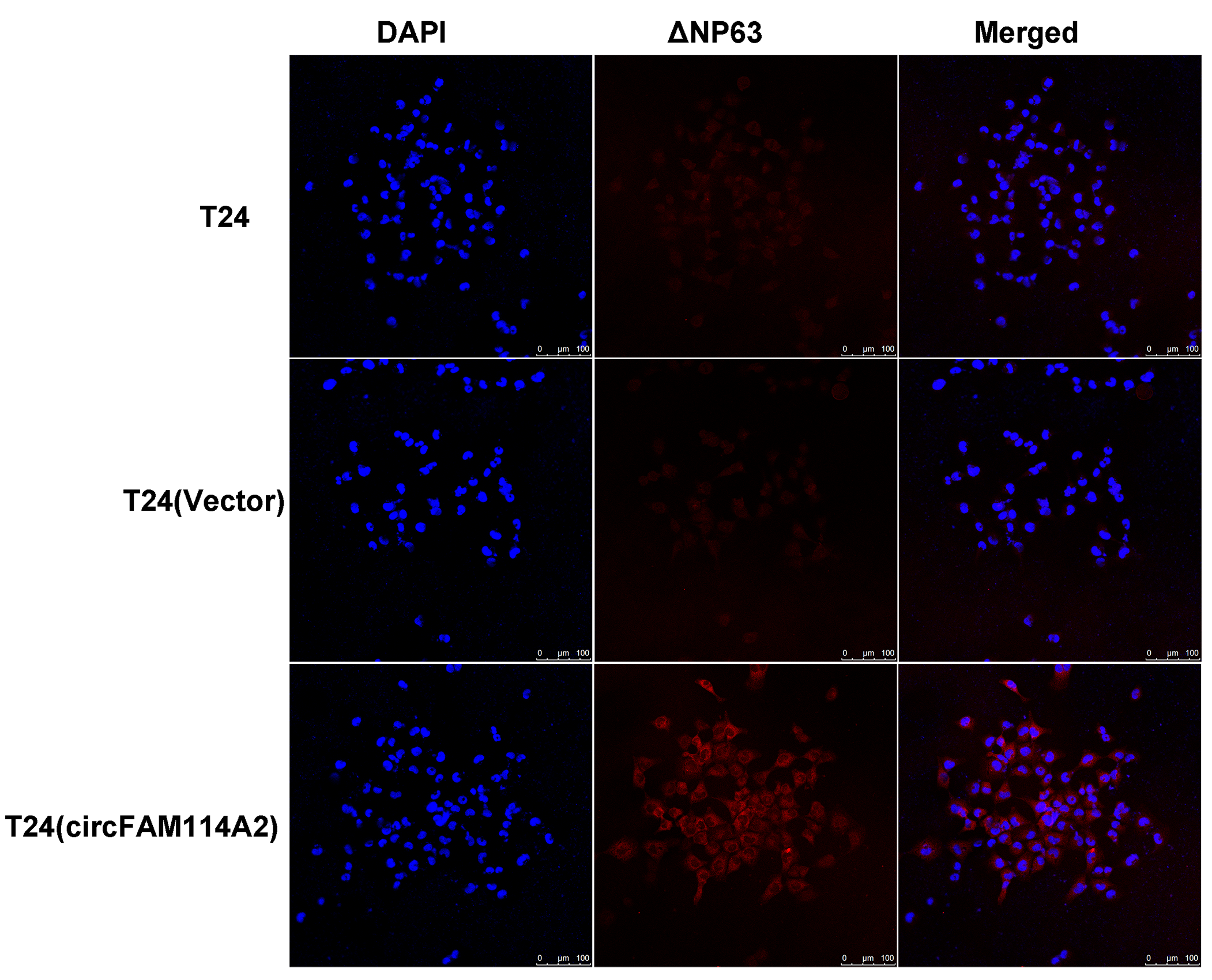

Supplement: Supplementary file 8 — The expression level of ∆NP63 in T24 cells. scale bars, 100um. [file 41419_2020_2226_MOESM8_ESM.tif]

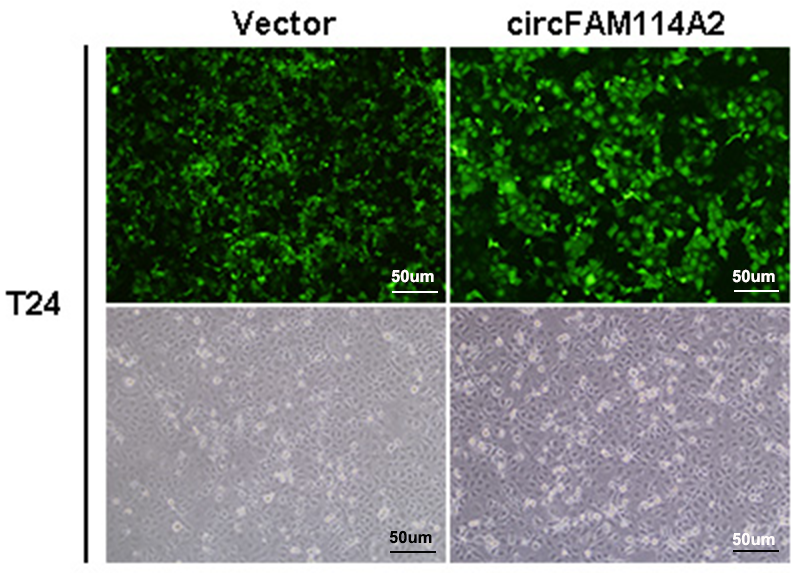

Supplement: Supplementary file 9 — The transfection efficiency of circFAM114A2 and Vector in T24 cells. scale bars, 50um. [file 41419_2020_2226_MOESM9_ESM.tif]

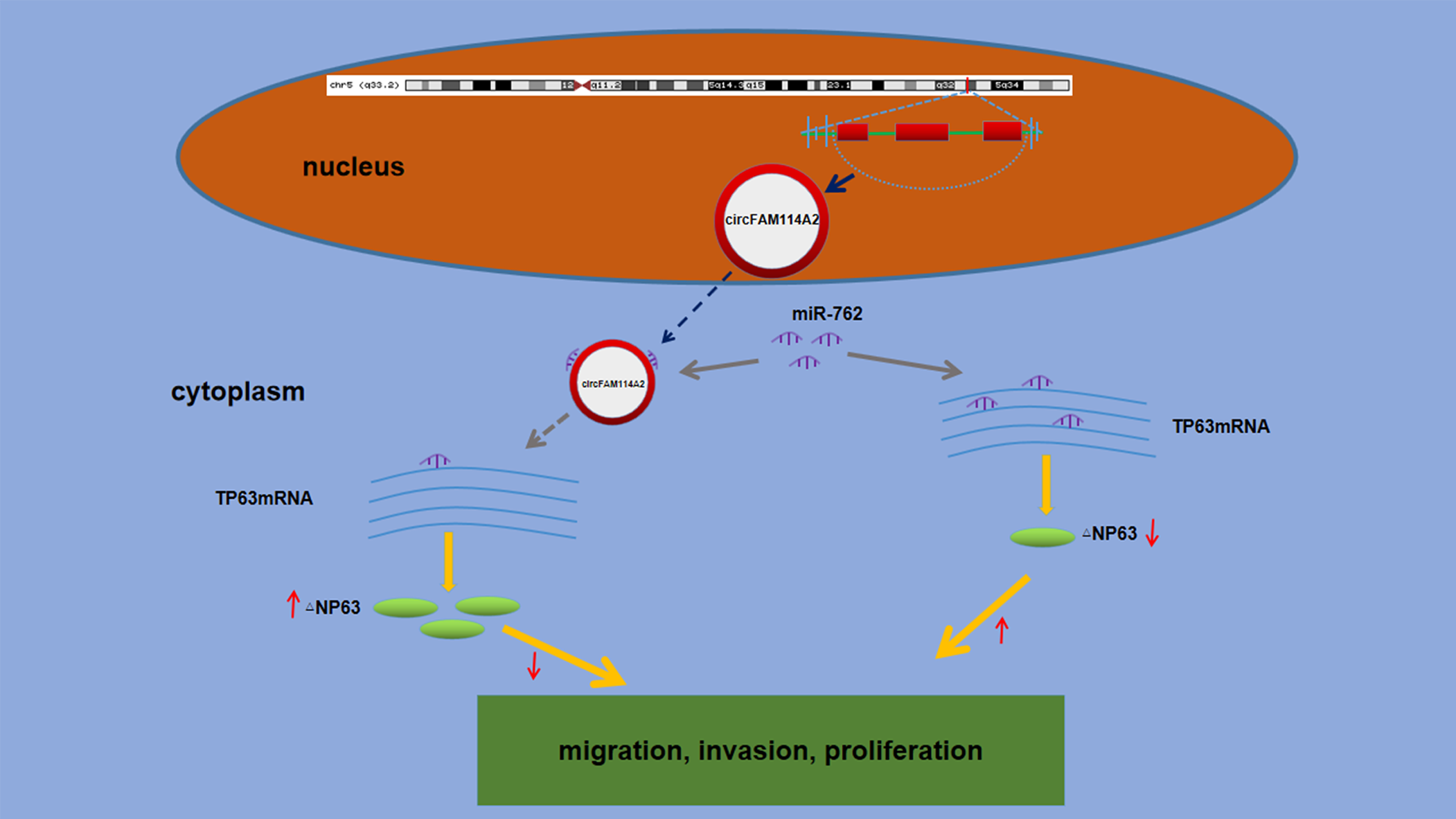

Supplement: Supplementary file 10 — The schematic diagram illustrates that circFAM114A2 serves as a ceRNA for miR-762, and inhibits the progression of UCB through the circFAM114A2/miR-762/∆ NP63 axis. [file 41419_2020_2226_MOESM10_ESM.tif]

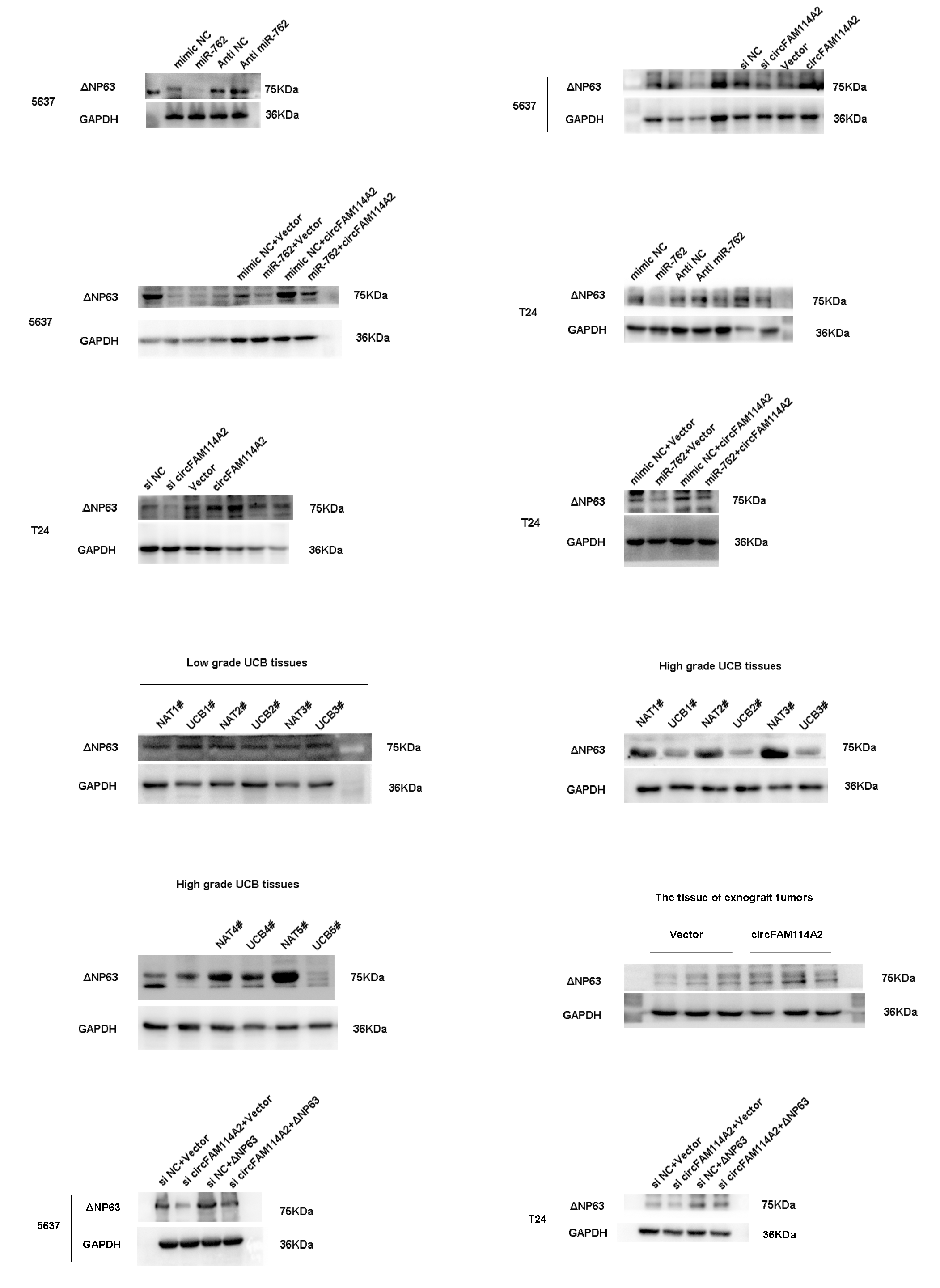

Supplement: Supplementary file 11 — The uncropped versions of western blots. [file 41419_2020_2226_MOESM11_ESM.tif]

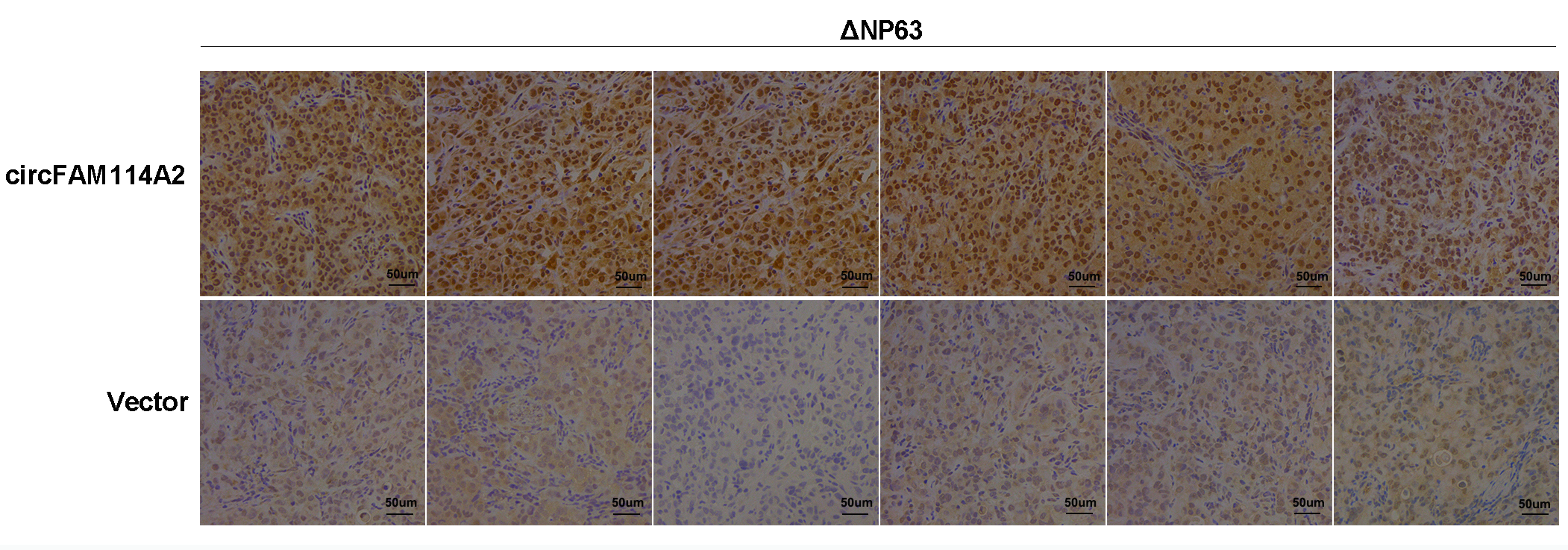

Supplement: Supplementary file 12 — The raw images of IHC of ∆ NP63 of each animal. [file 41419_2020_2226_MOESM12_ESM.tif]
